# Telemedicine and Geriatrics in France: Inventory of Experiments

**DOI:** 10.1155/2018/9042180

**Published:** 2018-09-17

**Authors:** A. A. Zulfiqar, A. Hajjam, S. Talha, M. Hajjam, J. Hajjam, S. Ervé, E. Andrès

**Affiliations:** ^1^Department of Geriatrics, University Hospital of Rouen, France; ^2^Research Unit EA 3072 “Mitochondrie, Stress Oxydant et Protection Musculaire”, Faculté de Médecine de Strasbourg, Université de Strasbourg (UDS), 4 rue Kirschleger, 67091 Strasbourg, France; ^3^Research Unit EA 4662 “Nanomédecine, Imagerie, Thérapeutiques”, Université de Technologie de Belfort-Montbéliard (UTBM), Belfort-Montbéliard, France; ^4^Service de Physiologie et d'Explorations Fonctionnelles, Hôpitaux Universitaires de Strasbourg (HUS), 1, porte de l'Hôpital, 67091 Strasbourg cedex, France; ^5^PREDIMED Technology, Mulhouse, France; ^6^Centre d'Expertise des Technologies de l'Information et de la Communication pour l'Autonomie (CenTich) et Mutualité Française Anjou-Mayenne (MFAM), Angers, France; ^7^Department of Internal Medicine, Diabetes, University Hospital of Strasbourg, 1, porte de l'Hôpital, 67091 Strasbourg cedex, France

## Abstract

Telemedicine is now in vogue, being deployed through computer and communication tools in various health fields, such as diabetology, nephrology, dermatology, neurology, and cardiology. With population ageing, geriatrics is coming into sharp focus. Telemedicine practices differ for home-based or institutionalized patients in long-term care homes. We take a look at telemedicine projects in France concerning the elderly.

## 1. Introduction

The European Union, notably France, is experiencing population ageing. This development will jeopardize the future balance of public finance, including that of healthcare. Life expectancy at birth continues to rise worldwide, and in Europe, it is approaching or exceeding 85 years for women and 80 years for men [1]. The quality of life in these extra years, experienced after age 80 or 85, is of increasing concern to authorities seeking to delay the onset of chronic diseases, senescence, functional decline, frailty, and loss of autonomy. The growing number of elderly people in the French population causes increased usage of the healthcare system. In 1997, people aged 60 and over represented almost one-third of the total medical expenditure and almost half of drug expenditure, while making up only 20% of the total population. People aged 65 and over have an average outlay of €3,000 per year, which is higher than that of the general population average of €1,800 per year [2]. These figures highlight the impact of the increasing number of elderly people in our country.

**Figure 1:**
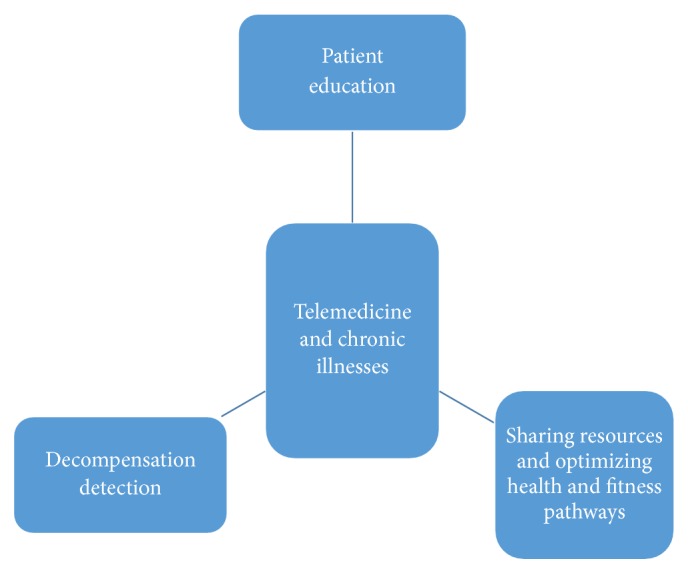
Telemedicine and chronic illnesses.

The development of information and communication technologies (ICTs) for the elderly is a promising venture. They create new opportunities to assist and care for elderly people at home or in specialized institutions, including nursing homes and hospitals. Grouped under the term* gerontechnology*, the first analyses of their contributions to this field appeared in the mid-1990s. Gerontechnology examines technology and ageing in order to improve living conditions and working environments, as well as medical care for dependent seniors [3] (Figure 1).

## 2. Definition and French Regulations for Telemedicine

According to the French Code of Public Health,* telemedicine* is defined as a form of remote medical practice using information and communication technologies. It allows healthcare professionals, with or without their patient's presence, to connect with one another, or with specialists involved in the patient's care [4, 5].

Telemedicine is a recent development in healthcare, enabled by technological progress and promoted by public authorities due to its potential possibilities, including improved accessibility to care, optimized use of professionals' time, improved collaboration between professionals, optimized care pathways, and revived innovation in therapeutic treatment plans [6–8].

Telemedicine has shown its effectiveness in the management of chronic diseases, such as heart failure and high blood pressure, among others. Monitoring patients with chronic diseases through the use of telemedicine is a way to optimize their care [9]. It also offers a better quality of life for elderly patients. Telemonitoring could lead to a significant reduction in the number of readmissions, which would consequently result in lower costs for society. These solutions also encourage the accurate collection of medical data to enable improved monitoring of patient health. The objectives are thus ambitious, ranging from improved rates of morbidity and mortality to decreased readmissions, improved quality of life, and reduced economic costs.

## 3. Method

We undertake a systematic review of related literature by searching electronic bibliographic databases in PubMed and Google Scholar and identifying studies published. The search was restricted to studies published in the era of French telemedicine projects or studies, results from elderly patients, and evaluation of home telemedicine programs implemented in France.

### 3.1. Telemedicine and Cardiology in the Elderly

Chronic heart failure affects more than 15 million Europeans, mostly the elderly [10]. Its development is marked by numerous episodes of acute heart failure, causing a high rate of repeated hospitalizations and significant medical expenditure. The SEDIC study, an educational, home-based study of patients with heart failure in Normandy, has researched the relevance of telemedicine devices since 2007 in a clinical, at-home study (SCAD). The structure of the study is organized around the patient and is based on the collection of data entered by the patient and sent to the educational center. It is part of a prospective, randomized, open-label, multicenter trial evaluating the impact of educational telemedicine aftercare for three months in patients over 65 and hospitalized for acute heart failure (LVEF <45%). The primary criterion is the number of days of hospitalization for acute heart failure after one year. The preliminary results at three months involved 73 patients: 35 randomly selected from those receiving traditional aftercare and 38 from those receiving telemedicine aftercare. Based on the main judgment criterion, there was not a significant difference. By the end of this study, 1,040 days of hospitalization from acute heart failure had been registered. The educational telemedicine aftercare was able to reduce this number, with 450 days in the telemedicine group compared to 590 days in the control group. Quality of life parameters improved equally in both groups. The study noted a significant decrease in cardiovascular mortality [11]. The average age for this sample is 76.8 years old, which is higher than those in other studies, such as the Tele-HF study's average age of 61 [12]. The patients from the SEDIC had better treatment plans than those in Tele-HF [12]. In the SEDIC study, 20% of patients had one or more cardiovascular events in three months, including death and hospital readmission, which was similar to the results found by Rich et al. [13]. Telemedicine can lower mortality rates: in fact, in the TEN–HMS study [14], patients who received telemedicine care lived longer than patients who received traditional aftercare. The meta-analyses are positive, with a reduction in morbidity and mortality rates in the telemedicine groups, according to COCHRANE reviews [15]. The results at three months in the SEDIC study, although statistically insignificant, reinforce the idea that telemedicine has a practical value in the aftercare of elderly patients with heart failure.

A study by Dary P aims to use at-home telemonitoring to evaluate the idea of optimizing treatments as a possible alternative to hospitalization [16]. 29 women and 54 men were included, with an average age of 78 years, 41% with a preserved ejection fraction and 59% with an altered ejection fraction. To evaluate the benefits of short-term telemonitoring, weight, blood pressure, and electrocardiograms were collected using a take-home telemedicine kit. The telemonitoring occurred directly between the patient and the cardiologist, so the treatment could be adapted in real time. Telemonitoring is not used as an alert system but rather aims to continually adapt the treatment according to the results of measured parameters. The results show an average weight loss of 2 kg (p<0.0001), linked to a 50% increase in the dosage of diuretics. During edema flare-ups, the rate of weight loss is a prognostic tool; if slow, it can predict hospitalization. Weight is an effective warning sign and powerful educational tool: it can predict short-term developments and indicate whether a situation is getting worse. The decrease in blood pressure is limited to 6 mmHg for systolic pressure (p = 0.002) and 7 mmHg for diastolic pressure (p <0.0001), allowing an increase in inhibitors of the conversion enzyme/sartans. The heart rate changes from 87 to 73 bpm (p <0.0001), which is more important in cases of atrial fibrillation. This is the second important criterion, after weight control that predicts the risk of hospitalization. One of the benefits of this method is the option to offer an alternative to hospitalization for 31 patients (37%)—specifically, management during edema flare-ups and emergencies, thus avoiding hospitalization. During the first 30 days after leaving the hospital, 4.4% of patients died, while 5% were readmitted for recurrence. This figure rose to 20% when all causes were included. In 2003, Goldberg et al. [17] published the WHARF study, which included the largest randomized multicenter sample and compared the value of telemonitoring aftercare to that of in-person aftercare. After 6 months, there was no difference in the rate or time frame of hospital readmissions (p = 0.28), but there was a significant reduction in mortality rates (p <0.003). The Telemonitoring to Improve Heart Failure Outcomes study (Tele-HF) [12] included patients who presented with decompensated heart failure in the last 30 days. The average age was 61. There was no notable difference between the two groups based on the criteria of mortality rate and hospitalization for any cause, nor on the secondary criteria of death, hospital readmission, and length of hospital stay.

The E-care telemonitoring project in Strasbourg falls under the category of telemedicine 2.0 [18]. It has been developed to optimize the home monitoring of heart failure patients. It detects situations where there is a risk of cardiac decompensation and rehospitalization, and it does this via a telemonitoring 2.0 platform. The E-care platform indicates when a patient's health status is worsening. These “warning alerts” are generated for any decompensation of a chronic disease (particularly heart failure) that may lead to hospitalization if not treated. The project used nonintrusive sensors deployed by the Department of Internal Medicine to record blood pressure, heart rate, oxygen saturation, and weight among selected elderly polypathological patients. These sensors are connected by Bluetooth and relay real-time physiological data about the patient's health status. The platform also includes a touchscreen tablet that is connected by Wi-Fi and a router or 3G/4G, making it possible to interact with the patient and advise on treatment, diet, and lifestyle. The E-care system includes a server that hosts the patient's data and a secure Internet portal that the patient and various hospital- and non-hospital-based healthcare professionals can connect to. The E-care telemonitoring platform was made available to patients as part of an experiment conducted by Strasbourg University Hospital [18]. Between February 2014 and April 2015, 175 patients benefited from the E-care platform [18]. During this period, the E-care platform was used on a daily basis by patients and healthcare professionals, according to a defined protocol of use specific to each patient. The mean age of these patients was 72 years and the ratio of men to women was 0.7. The patients suffered from multiple concomitant diseases and had a mean Charlson index of 4.1. The main medical history of the 175 patients included were dominated by heart failure in more than 60% of the subjects, anemia in more than 40%, atrial fibrillation in 30%, type II diabetes in 30%, and chronic obstructive pulmonary disease in 30%. During the study, 1,500 measurements were taken for these 175 patients, which resulted in the E-care system generating 700 alerts for 68 patients [18]. Analysis of the warning alerts showed that the E-care platform automatically and nonintrusively detected any worsening of the patient's health, particularly for cardiac decompensation. Indeed, it was in this aspect that the system yielded the best sensitivity, specificity, and positive and negative predictive values, at 100%, 72%, 90%, and 100%, respectively. The E-care platform also demonstrated its ability to detect deterioration in health status via the multiple diseases present in the patients studied, with sensitivity, specificity, and positive and negative predictive values of 100%, 30%, 89%, and 100%, respectively. The level of caregiver satisfaction was considered adequate.

Other studies have been conducted like the COMPAS study as COMPArative follow-up Schedule with home monitoring, with the firm Biotronik, involving monitoring cardiac pacemakers with 538 patients randomized into 2 branches (teletransmission versus traditional surveillance). The study showed the safety of patient monitoring through telecardiology over a period of 18 months [19]. SETAM study is a study about telemedicine in rhythmology. It was a randomized study (strategy of early detection and active management of supraventricular arrhythmia with telecardiology) in 2 groups of patients wearing cardiac pacemakers. It showed that this stimulated early detection of occurrences, with 66% reduction in hospitalizations linked to atrial arrhythmias and stroke prevention. The SETAM study is a prospective, multicentered, randomized study, the objective of which is to assess the benefits of telecardiology in screening and implementing therapeutic strategies in atrial fibrillation. The study showed that telecardiology allows for faster diagnosis and treatment of patients with atrial arrhythmias as well as a reduction of the load in atrial fibrillation within 9 months of monitoring patients in sinus rhythm at the time of inclusion with a score of CHA2DS2-VASc>=2. 595 patients have been included in the General Center Hospital in France in a period of 12.8 +/- 3.3 on average. Average age = 79 +/- 8 years old [20].

Cardiauvergne ensures the monitoring and coordination of care for 2000 patients of heart failure throughout the Auvergne region, older than 60 years of age, through sharing information via a digital medical file. One single sensor is assigned to the patient: a scale with a teletransmitter. Nurses are equipped with a smartphone for access, just as laboratories and pharmacists access patient files [21].

### 3.2. Telemedicine and Dermatology for the Elderly

In Aquitaine, an experiment on teleconsultation was conducted from September 2012 to September 2013 involving six nursing homes. 19 patients were included with an average age of 82.4. 51 teleconsultations were performed, looking at aftercare for bedsores (57.9%), trophic vascular ulcers (26.3%), and trauma wounds (15.8%). Telemedicine significantly improved the wound healing process and reduced expenditure on bandages by reducing the rate of dressing changes (p <0.005). The stakes of telemedicine are high in geriatrics, especially as regards aftercare for elderly people with chronic conditions requiring repeated trips and admissions to the hospital. This work showed an excellent level of aftercare with a rate of recommendations close to 100%. The information and assistance available during teleconsultations provided notable theoretical and practical training to carers in nursing homes. On the economic side, this study showed that remote consultations were able to eliminate the need for in-person consultations or hospitalization for 79% of the patients included [22]. Telemedicine could also play an essential role in improving the isolation and education of carers in nursing homes.

In 2009, at the CHU Besançon Dermatology Department's outpatient treatment center for chronic wounds, 240 patients received 820 bandages from outpatient services. Most of these cases involved frail, elderly patients with limited mobility. The Dermatology Department decided to set up a telemedicine service to provide remote follow-up care and diagnostics for the wounds. The objectives were to improve patient aftercare by limiting the number of consultations at CHRU Besançon's treatment center, reduce the cost of care, and offer local hospitals, private practices, and nursing homes a structured support network [23]. In 2003, a study by the American Telemedicine Association (ATA) already reported 62 experiments in 37 different USA states using telemedicine for dermatology [24]. There are two methods of support defined by the ATA:

- *Store-and-forward teledermatology* involves an expert consultant communicating remotely with the patient's dermatologist and using digital images and clinical information to make a diagnosis.
- *Live interactive teledermatology* is a teleconsultation between the patient and health professionals using videoconferencing equipment [23, 24].

This method of care is currently widespread, as shown by the Télégéria network. Télégéria is one of only a few teleconsultation experiments in dermatology and has completed approximately 100 consultations since the project's launch in 2004 [25].

An experimentation through tele-expertise for treating chronic wounds in elderly subjects residing in nursing home was conducted in the Haute-Vienne region. The goal is to avoid moving elderly patients often with reduced mobility as well as the costs generated by ambulance transportation. Of the 40 nursing homes in Haute-Vienne invited to share, 22 committed to it, but only the first 10 responses were accepted. Between April 15, 2010, and April 15, 2012, digital photographs of 34 patients selected by 10 nursing homes were sent to the messaging service specifically made for the Limoges University Hospital. The average number of teletransmissions by these nursing homes was 3.4 in 2 years. These 34 cases accounted for 26 chronic wounds in 24 patients. There were 10 pressure sores, 2 perforating foot ulcers, and 14 leg ulcers. Twenty roundtrip ambulatory commutes were avoided. Tele-expertise has allowed 20 patients to receive better care for chronic wounds without moving to a hospital [26]. Other experiments in telemedicine in the field of chronic wounds exist in France such as CICAT in Languedoc-Roussillon (now Occitanie) and TELAP in Normandy. Thus, the service of telemedicine Domoplaies has allowed a rate of healing of complex wounds and/or chronic conditions at half the cost if compared to typical treatment according to a medico-economic analysis by the CICAT-Occitanie network [27].

### 3.3. Telemedicine and Nursing Homes

One of telemedicine's aims is to spread to nursing homes.

Among the first telemedicine experiments in France involving nursing homes is Télégéria. Télégéria is a system offering teleconsultations, video consultations with experts, and tele-assistance for the elderly in geriatric hospitals and nursing homes. Being a telemedicine network founded in 2004, Télégéria gives patients (who are living in nursing homes or hospitalized at the geriatric hospital Vaugirard Gabriel-Pallez) the benefit of expert advice through teleconsultations with Georges Pompidou European Hospital (HEGP, AP-HP). The network is based on the observation of polypathology among the elderly, which requires advice from multidisciplinary specialists. The medical objective is to evaluate the interest and potential of using telemedicine for clinical sessions in more than 20 areas of expertise, including orthopedics, dermatology, care of pressure ulcers, vascular medicine, palliative care, pneumology, neurology, and urology, as well as sessions between hospital geriatricians and coordinating physicians from nursing homes.

Dr. Pierre Espinoza is a coordinator of the Télégéria project for remote geriatrics aiming to connect retirement homes with hospital specialists through telemedicine. A 15-month activity report was able to identify 700 telemedicine sessions that involved the following areas in the following distributions: orthopedics (35%), cardiology and cardiac and vascular ultrasounds (32%), dermatology (17%), neurology (4%), and geriatrics (2%). Télégéria's regional deployment in Ile-de-France plans to expand to 30 nursing homes connected to two major hospitals in the 75^th^ and 95^th^ districts [28].

Other programs have emerged, such as the TELEHPAD program aimed at facilitating access to care in rural areas. Patients, including both nursing home residents and the general local population, access teleconsultations thanks to rooms in the nursing homes directly connected to general and psychiatric hospitals. Thus, they can benefit from teleconsultations in geriatric medicine, psychiatry, dermatology, cardiology, and neurology. TELEFIGAR is another program aimed at offering nursing home residents the option of teleconsultations with experts in geriatrics, neurology, dermatology, and diabetology. It brings together the University Hospital of Rennes, the Regional Geriatric Center of Chantepie, three nursing homes, and the* Réseau Diabète 35 *organization.

Comprehensive geriatric assessment (CGA) is equally feasible in nursing homes. This was shown by a retrospective study in Bordeaux that looked at teleconsultations carried out in 39 nursing homes in the Gironde and Dordogne regions [29]. 304 residents benefited from telemedicine for the management of complex conditions, including psychological disorders, Alzheimer's or related diseases, chronic wounds, and psychopathologies, as well as conditions requiring palliative care. The data collected included the average age of the residents, ADL, MMSE, CIRS-G, and the number and type of scheduled medications taken each day during hospitalization. 500 teleconsultations were analyzed, with 28.4% concerning psychobehavioral disorders, 27.8% concerning complex chronic wounds, 9.2% concerning dermatology, 19% concerning psychopathologies, and 2.8% concerning palliative care. Teleconsultation procedures managed to prevent hospital transfers in 378 cases (75.6%), and the therapeutic treatment plans were optimized in 351 cases (70.2%). This preliminary study showed that it is possible to conduct CGAs through teleconsultations. Few studies have been conducted concerning telemonitoring in nursing homes [30]. However, another study by Grabowski and O'Malley, including randomly selected patients [31] from 11 nursing homes, found similar results: there was a significant reduction (11.3%) in the number of hospitalizations among residents receiving telemedicine care.

A study in Toulouse analyzed the reasons for teleconsultations between the Gaubert nursing home in Toulouse and the geriatric ward of CHU Purpan of Toulouse [32]. This was a retrospective, descriptive study involving 60 residents over a period of 19 months, during which 28 teleconsultations took place with 16 different residents. Of the 28 consultations, 18 concerned the diagnostic and treatment plans of patients with severe dementia. One consultation concerned a patient's agitation at being bathed, while others were about the management of patients with aberrant motor behaviors and nocturnal ambulation. Four consultations dealt with dermatological issues, like ulcers. Two consultations concerned iatrogenic risk assessment in polymedicated patients, and two concerned patients who have experienced falls. Mathieu-Fritz [33] conducted a study with the Télégéria project concerning teleconsultations between the HEGP and a geriatric hospital from June 2009 to February 2010. The 333 consultations involved 16 areas of expertise: 35% concerned orthopedics and 16% concerned dermatology. The average age of a Télégéria participant is 85.7. Teleconsultations avoid unnecessary transport, which can be challenging for frail patients [34]. Involving the patient and their family in the professional exchange through teleconsultation reinforces their commitment to the patient's treatment plan. The tele-consulting project in nursing home was initiated by ARS Lorraine alongside the CHRU of Nancy and the firm Télésanté Lorraine. The measure is part of the experiment “Elderly Individuals at Risk of Losing Independence” or PAERPA. The first teleconsultations in nursing home in Lorraine were inaugurated on June 24, 2014. Two nursing homes were initially included (the Park Residence and the Saint-Joseph Residence), and then 3 other nursing homes joined their ranks, bringing the number to 5. Five fields of medical query have been targeted with cardiovascular and lung diseases, geriatric psychiatry, dermatology, medicine-related illnesses, and palliative care. In total, 60 teleconsultations were conducted for 41 residents who were on average 87.2 years old [35].

A “telemedicine, gerontology, and rurality” project conducted in the Gorre Valley focuses on 9 nursing homes in the region of Limousin (675 residents). The goal is to decrease the number of hospitalizations and transports of elderly residents in order to improve the care they receive. Those leading the project are conducting a medico-economic assessment with an estimated 25% decrease in healthcare expenditures [36].

Telemedicine has recently been developed in response to epidemiological, demographical, and economical problems in the French Healthcare system and telepsychiatry is no exception to this rule but few studies have evaluated its applicability and acceptability when applied to the elderly. The Du Rouvray Center's psychiatric team at the Rouen University Hospital is equipped with a telemedicine service for psychiatric consultation in retirement homes in the Seine-Maritime. Since the start of the program, activity has gone from about one hundred external consultations per year to one hundred per month [37].

Other studies have been conducted concerning telemedicine and oral health among patients in nursing homes. The oral health of residents in communal medical institutions is alarming. The few studies carried out show that almost half of the elderly people living in long-term care facilities have not had consultations with a dentist in over five years [38]. The rate of appointments with dentists among the residents of these facilities is less than 25% of that of senior citizens living at home [39]. The primary objective of the e-DENT project is to validate the use of telemedicine in dentistry, mainly for residents in nursing homes [40]. This is the first experiment of its kind in France. It will include 400 teleconsultations in 8 nursing homes managed by the Alzès Central Hospital and 200 teleconsultations in 4 nursing homes managed by the Thau Basin Central Hospital. These 600 patients will have the benefit of 2 remote consultations each, at 6-month intervals. In nursing homes, remote oral consultations will finally enable general health checkups for each resident. Oral health is often neglected, despite recommendations from the French National Authority for Health (HAS) and health insurers that senior citizens in care have an oral checkup and at least one dentist appointment every year. Dental telemedicine lowers the cost of these checkups. A specially trained nurse uses a SoproCare camera with fluorescent light to take videos and photos of the inside of the patient's mouth, allowing for easy detection of cavities and gum disease. A study in Limousin entitled the “TELEDENT study” examined the same issue. This study was conducted by Limoges University Hospital and Guéret Hospital, with the goal of using telemedicine to prevent tooth problems in the elderly. In total, 235 patients were examined. The average age was 84.4, ± 8.3 years, and 59.1% of the subjects were women. A total of 128 (55.4%) patients had a dental disease. The sensitivity of the remote sensing was 93.8% for the diagnosis of dental disease (95% confidence interval 90.7-96.9), and the specificity was 94.2% (95% CI 91.2-97.2). Of the 128 cases of dental disease identified by remote sensing, 6 (4.8%) were false positives. Remote sensing assessments were faster than face-to-face exams, taking 12 and 20 minutes, respectively [41].

The LAAS is interested in issues concerning the surveillance of elderly individuals. The PROSAFE project was conducted as part of a collaboration between the LAAS and EDF R and D during the nineties. It is a nonintrusive surveillance support system for elderly and handicapped individuals. The system has been the subject of tests on 3 patients for 8 months (tele-surveillance of the patient in room) inside the Charron nursing home (Charente Maritime). It was then installed in several apartments (residential homes for the elderly in Orleans) to monitor a single person living alone [42].

### 3.4. Telemedicine in Geriatric Home Care

Telemedicine projects in geriatric home care are beginning to multiply. The ESOPPE project, conducted in Corrèze, France, is unique in Europe. It is an economic, social, and environmental assessment of home automation and advanced remote assistance (DTA) among the elderly living at home with limited autonomy. The program included 194 adults aged 65 and over living at home and registered on a list of frail elderly people. Participants were uniformly asked about their history of falls during the year prior to their most recent health examination. The recall period was one year. 77 (40.5%) elderly fell at home, 29 (30.9%) in the exposed group and 48 (50.0%) in the unexposed group. The use of light path coupled with tele-assistance was significantly associated with the reduction in falls at home (p value = 0.0012). There was also a greater reduction in postfall hospitalization rate among exposed group (p value = 0.009). In fact, a 30% decrease was noted in falls among people using the home automation and remote assistance package. In terms of falls, DTA considerably improves outcomes, both in number and severity. At the end of the experiment, elderly people with DTA were significantly less depressed than the control subjects. In terms of dependence, the trend seems positive, with a marked improvement according to the French index for measuring independence (GIR), but this has yet to be validated over a longer period. During the experiment, there were no known cancellations of the DTA package [43].

Since autumn of 2015, 268 patients over the age of 65 from Limousin and Loir-et-Cher have been receiving aftercare from the Geriatric Department and the Chronic Disease Department of the Limoges Hospital without actually going to the hospital. Their care depends on the home nurses who examine them using devices connected to the Internet. Limoges University Hospital is a participant in the iCare project. Being one of a kind in Europe, this pilot research project evaluates the effectiveness of at-home, medical telemonitoring for elderly people with chronic diseases. Their objective is to demonstrate that telemonitoring of chronic diseases in elderly patients prevents decompensation, loss of balance, and unexpected hospitalization.

The study was conducted over a period of 12 months with 500 senior citizen volunteers. Among them, some had the benefit of telemonitoring through biometric sensors installed in their homes. These sensors, which do not need to be worn on the body, monitor constants, such as blood pressure, blood glucose, weight, blood oxygenation level, and temperature. Each day, a transmitter will securely send the data to the patient's attending physician, private nurse, and a CHU geriatric specialist simultaneously [44]. Franco et al. worked on a study of elderly people with Alzheimer's disease who had telemonitoring in their homes [45]. The system allowed them to detect nycthemeral rhythm drifts from location data.

One should mention the DETECT study, which looks at telemedicine for the management of psychobehavioral disorders. The study is sponsored by Toulouse University Hospital, and the coordinating investigator is Professor Soto. This is a randomized, open-label, multicenter intervention study, with a control group receiving usual care and an intervention group receiving remote expert consultations. 20 nursing homes are involved. The main objective is to evaluate the acceptability of expert teleconsultation, with the secondary goals of tracing and comparing specific parameters in the two nursing home groups, such as the rate of hospitalizations and consultations for behavioral and psychological symptoms of dementia (BPSD), the rate of prescriptions for neuroleptic and psychotropic drugs, patient care costs, and patient quality of life [46].

The GERONTOACCESS study (2015-2017) revolves around the use of telemedicine in rural gerontology in the Saint-Laurent-sur-Gorre commune. It includes 9 nursing homes and 428 seniors in Limousin, with the main objective of reducing unscheduled hospital admissions. 1,000 incidents per year were counted, including those from DETECT [47]. The results are currently being analyzed.

SALVEO is a smart system of monitoring and tele-assistance for the elderly living alone at home. It analyzed collected data of environmental sensors like “movement, temperature, door contact” deployed on site. This server enables sending alerts to family members or to nursing assistants. The occurrences detected by this system are failure to wake up, falls, absence of movements, abnormal temperatures inside housing, sleep disturbance, time length of kitchen and bathroom use, and the individual's rate of mobility [48].

The project OURSES, or “Supplying Rural Use of Services by Satellite”, is a project of the competitive cluster “Aeronautics, Space and Embedded Systems” in the Midi-Pyrénées and Aquitaine regions. This project aims to highlight the benefits of communication technologies by satellite for direct surveillance of isolated individuals in rural areas who do not have means to access high-speed networks. This is a telemedicine system based on remote monitoring, to remotely monitor the electrocardiogram in real time. The system analyzes the signal from the ECG sensor that the patient wears. The doctor can be alerted if the system detects a fault or can remotely analyze all stored data. It also seeks to follow behavioral deviations of the elderly subject in the night thanks to motion detectors activated in room. Alerts are sent to the remote doctor's PC when an unusual situation has been detected. The OURSES system was the subject of a clinical assessment in “Tibiran-Jaunac” in the middle of a rural region in the Hautes-Pyrenées [49].

And finally, we can evoke the HOMECARE project aims at testing and qualifying, in terms of operations, a complete system of tele-surveillance for elderly individuals suffering from Alzheimer's disease. A surveillance platform has been institutionally activated at the local hospital in Caussade, based on the collection of location data from a network of hybrid sensors. The doctor and nursing staff receive alarm notifications. This consultation has to be done with a computer interface and presupposes remote access (i.e., tele-surveillance system based on a network of multisensory sensors) [50].

We summarize these projects in Tables 1 and 2

## 4. Limit

The goal of telemedicine is not to replace doctors, but rather to assist them on a daily basis to simplify contact and help diagnosis by reducing the loss of time (transport, for example). Beyond reducing the problem of medical deserts, the deployment of telemedicine in France would optimize the use of doctors' time, regularly faced with the withdrawal of patients and cancellations of appointments at the last moment.

On the other hand, telemedicine consultations are currently not supported by social security. The price of the consultation is therefore the responsibility of the patient, or his complementary health. Ultimately, telemedicine must be accompanied by a satisfactory management to seduce patients. In addition, the implementation of these devices remains very expensive, whether in the acquisition or maintenance, but also and especially in the training of professionals (and patients) who will use them. In addition, there is still a concern about the transmission of health data, which can be easily manipulated. The CNIL, however, ensured its control of these health data. These practices must also be very well supervised. One of the keys to this success is also training health professionals in these innovative techniques as well as facilitating access to reimbursement for telemedicine.

## 5. Conclusion

The avenues for telemedicine applications are gradually opening up to geriatrics, and projects should increase in the years to come. There are multiple forces driving this, but the preservation of quality of life for seniors at home and in retirement homes is an especial priority. Currently, thanks to the E-CARE platform, our team is developing a system for telemonitoring geriatric risk among nursing home residents with the aim of reducing preventable hospitalizations in emergency departments and preventing decompensation of patients with geriatric risk factors.

## Figures and Tables

**Table 1:** Telemedicine projects in nursing homes.

**Telepsychiatry between CHSR [downtown hospital] Rouvray (CHU [University Hospital] Rouen) and Gournay nursing home**	Tele-consultations	One hundred monthly consultations	Psychiatry

**Télésanté [e-Health] Lorraine/ARS [regional health agency] Lorraine**	Tele-consultations	60 tele-consultations for 41 residents	Cardiology-Gerontopsychiatry-Dermatology-Iatrogenesis-Palliative Care

**“Telemedicine, gerontology, and rurality” project of the Gorre valley (Limousin region)**	Tele-consultation	9 nursing homes included (675 residents), project in progress	Geriatrics

**Chronic wounds telemedicine nursing home in Haute Vienne**	Tele-expertise	10 nursing homes involved, photographs of 34 residents sent via digital mail over a period of 2 years	Dermatology

**PROSAFE Project**	Remote surveillance	Experiment on 3 elderly patients during 8 months in the nursing home of Charron (Charente Maritime)	Geriatrics

**OURSES project**	Remote surveillance	Clinical evaluation (ECG) in a nursing home (Tibiran-Jaunac (Hautes-Pyrénées))	Geriatrics

**Aquitaine Chronic Pressure Sensors Télésanté Project (CHU Bordeaux) **	Tele-consultation	6 nursing homes involved, 19 residents included over a period of one year	Dermatology

**Télégéria [remote geriatrics] (between nursing homes/Vaugirard Gabriel-Pallez Hospital and Georges Pompidou European Hospital)**	Tele-consultation, tele-expertise hotline	Activity report at 15 months = 700 sessions	Orthopedics-Dermatology-Vascular Medicine-Palliative Care-Pulmonology-Neurology-Urology

**TELEHPAD **	Tele-consultation	Breton telemedicine program, initiated by the French Mutuality of Côtes-d'Armor, project in progress	

**TELEFIGAR**	Tele-consultation and tele-expertise	An ongoing project	Geriatrics, Neurology, Dermatology, Diabetology

**Standardized gerontology evaluation project via telemedicine in nursing home (Bordeaux University Hospital)**	tele-consultation	39 nursing homes involved, 304 residents, 500 tele-consultations performed	Geriatrics

**e-DENT project**	Tele-consultation	400 tele-consultations in 8 nursing homes in CH [Downtown Hospital] Alès and 200 tele-consultations in 4 nursing homes in CH Thau	Dentistry

**TELEDENT (between CHU Limoges and nursing home of Guéret Hospital)**	Tele-consultation	235 patients examined, with 128 residents with a dental problem	Dentistry

**DETECT study (CHU Toulouse)**	Tele-expertise and remote surveillance	Project in progress, 20 nursing homes included	Geriatrics

**GERONTOACCESS study**	Tele-consultation	428 residents of 9 nursing homes involved, project in progress	Geriatrics

**TELEPAL**	Tele-expertise and tele-consultation	Involvement of 51 nursing homes in the area of GCS [Geriatric Care Services] Geriatrics = Valenciennes, Saint Amand, Denain, Le Quesnoy, and 2 mobile palliative care teams	Palliative care

**Table 2:** Outpatient telemedicine projects.

**ECARE project**	**Remote surveillance **	**175 patients with heart failure, 700 alerts in 68 patients**	**Cardiology **

**COMPAS study**	Remote surveillance	It revealed security in monitoring patients with pacemakers	Cardiology
**SETAM study**	Remote surveillance	2,595 patients included out of 57 general hospitals; it revealed treating patients with atrial arrhythmias as soon as possible	Cardiology
**Cardiauvergne **	Remote surveillance	Halving of mortality and nearly 2/3 the rate of re-admissions on the first 538 patients included	Cardiology
**DOMOPLAIES (CICAT-Occitanie/TELAP Normandy)**	Tele-expertise	14,000 tele-consultations for 4,500 patients, with improved healing rate and reduced costs	Dermatology
**SALVEO**	Helpline	Use of environmental sensors	Geriatrics
**HOMECARE project**	Remote surveillance	Monitoring of patients with Alzheimer's disease, thanks to the use of a hybrid sensor network, experimentation at the local hospital of Caussade	Geriatrics
**SEDIC study**	Remote surveillance	Home experimentation in elderly patients with heart failure; it showed a reduction in cardiovascular mortality and number of days of hospitalization	Cardiology
**Study by Dary, P. et al.**	Remote surveillance	Optimization of treatments in 83 heart failure patients at home; it revealed weight loss, a 26% reduction in hospitalizations, and a reduction in heart failure deaths	Cardiology
**Project of the Chronic Wounds Outpatient Treatment Center at CHRU Besançon**	Tele-consultations-Helpline	820 outpatient wound dressings and 240 patients followed up	Dermatology
**ESOPPE project**	Helpline	Experiment on 100 elderly people at home; it reveals a 30% reduction in falls	Geriatrics
**e-COBAHLT/ICARE Project**	Remote surveillance	Ongoing experiment on 536 elderly patients, in collaboration with Limoges University Hospital	Geriatrics
